# Identification of ADHD risk genes in extended pedigrees by combining linkage analysis and whole-exome sequencing

**DOI:** 10.1038/s41380-018-0210-6

**Published:** 2018-08-16

**Authors:** Jordi Corominas, Marieke Klein, Tetyana Zayats, Olga Rivero, Georg C. Ziegler, Marc Pauper, Kornelia Neveling, Geert Poelmans, Charline Jansch, Evgeniy Svirin, Julia Geissler, Heike Weber, Andreas Reif, Alejandro Arias Vasquez, Tessel E. Galesloot, Lambertus A. L. M. Kiemeney, Jan K. Buitelaar, Josep-Antoni Ramos-Quiroga, Bru Cormand, Marta Ribasés, Kristian Hveem, Maiken Elvestad Gabrielsen, Per Hoffmann, Sven Cichon, Jan Haavik, Stefan Johansson, Christian P. Jacob, Marcel Romanos, Barbara Franke, Klaus-Peter Lesch

**Affiliations:** 1 Department of Human Genetics, Donders Institute for Brain, Cognition and Behaviour, Radboud University Medical Center, Nijmegen, The Netherlands; 2 K.G. Jebsen Centre for Neuropsychiatric Disorders, Department of Biomedicine, University of Bergen, Bergen, Norway; 3 Division of Molecular Psychiatry, Clinical Research Unit on Disorders of Neurodevelopment and Cognition, Center of Mental Health, University of Würzburg, Würzburg, Germany; 4 Department of Molecular Animal Physiology, Donders Institute for Brain, Cognition and Behaviour, Radboud University Medical Center, Nijmegen, The Netherlands; 5 Laboratory of Psychiatric Neurobiology, Institute of Molecular Medicine, I.M. Sechenov First Moscow State Medical University, Moscow, Russia; 6 Department of Child and Adolescent Psychiatry, Psychosomatics and Psychotherapy, Center of Mental Health, University Hospital of Würzburg, Würzburg, Germany; 7 Department of Psychiatry, Psychosomatics and Psychotherapy, Center of Mental Health, University of Würzburg, Würzburg, Germany; 8 Department of Psychiatry, Psychosomatic Medicine and Psychotherapy, University Hospital Frankfurt, Frankfurt am Main, Germany; 9 Department of Psychiatry, Donders Institute for Brain, Cognition and Behaviour, Radboud University Medical Center, Nijmegen, The Netherlands; 10 Department of Cognitive Neuroscience, Donders Institute for Brain, Cognition and Behaviour, Radboud University Medical Center, Nijmegen, The Netherlands; 11 Department for Health Evidence, Radboud Institute for Health Sciences, Radboud University Medical Center, Nijmegen, The Netherlands; 12 Biomedical Network Research Center on Mental Health (CIBERSAM), Institute of Salud Carlos III, Madrid, Spain; 13 Department of Psychiatry and Forensic Medicine, Universitat Autònoma de Barcelona, Barcelona, Catalonia Spain; 14 Department of Psychiatry, University Hospital Vall d’Hebron, Universitat Autònoma de Barcelona, Barcelona, Catalonia Spain; 15 Psychiatric Genetics Unit, Group of Psychiatry, Mental Health and Addiction, Vall d’Hebron Research Institute (VHIR), Universitat Autònoma de Barcelona, Barcelona, Spain; 16 Department of Genetics, Microbiology and Statistics, Faculty of Biology, University of Barcelona, Barcelona, Catalonia Spain; 17 Institut de Biomedicina de la Universitat de Barcelona (IBUB), Barcelona, Catalonia Spain; 18 Instituto de Investigación Biomédica en Red de Enfermedades Raras (CIBERER), Institute of Salud Carlos III, Madrid, Spain; 19 Institut de Recerca Sant Joan de Déu (IR-SJD), Esplugues de Llobregat, Catalonia Spain; 20 K.G. Jebsen Center for Genetic Epidemiology, Department of Public Health, NTNU, Norwegian University of Science and Technology, Trondheim, Norway; 21 HUNT Research Centre, Department of Public Health, Norwegian University of Science and Technology, Levanger, Norway; 22 Institute of Human Genetics, University of Bonn, Bonn, Germany; 23 Department of Genomics, Life&Brain Center, University of Bonn, Bonn, Germany; 24 Institute of Medical Genetics and Pathology, University Hospital Basel, Basel, Switzerland; 25 Department of Biomedicine, University of Basel, Basel, Switzerland; 26 Institute of Neuroscience and Medicine (INM-1), Research Center Jülich, Jülich, Germany; 27 Division of Psychiatry, Haukeland University Hospital, Bergen, Norway; 28 Center for Medical Genetics and Molecular Medicine, Haukeland University Hospital, Bergen, Norway; 29 K.G. Jebsen Centre for Neuropsychiatric Disorders, Department of Clinical Science, University of Bergen, Bergen, Norway; 30 Department of Neuroscience, School of Mental Health and Neuroscience, Maastricht University, Maastricht, The Netherlands

**Keywords:** Genetics, ADHD

## Abstract

Attention-deficit/hyperactivity disorder (ADHD) is a common neurodevelopmental disorder with a complex genetic background, hampering identification of underlying genetic risk factors. We hypothesized that combining linkage analysis and whole-exome sequencing (WES) in multi-generation pedigrees with multiple affected individuals can point toward novel ADHD genes. Three families with multiple ADHD-affected members (*N*_total_ = 70) and apparent dominant inheritance pattern were included in this study. Genotyping was performed in 37 family members, and WES was additionally carried out in 10 of those. Linkage analysis was performed using multi-point analysis in Superlink Online SNP 1.1. From prioritized linkage regions with a LOD score ≥ 2, a total of 24 genes harboring rare variants were selected. Those genes were taken forward and were jointly analyzed in gene-set analyses of exome-chip data using the MAGMA software in an independent sample of patients with persistent ADHD and healthy controls (*N* = 9365). The gene-set including all 24 genes together, and particularly the gene-set from one of the three families (12 genes), were significantly associated with persistent ADHD in this sample. Among the latter, gene-wide analysis for the *AAED1* gene reached significance. A rare variant (rs151326868) within *AAED1* segregated with ADHD in one of the families. The analytic strategy followed here is an effective approach for identifying novel ADHD risk genes. Additionally, this study suggests that both rare and more frequent variants in multiple genes act together in contributing to ADHD risk, even in individual multi-case families.

## Introduction

Attention-deficit/hyperactivity disorder (ADHD) is a multifactorial neurodevelopmental disorder, characterized by age-inappropriate inattention, hyperactivity, and increased impulsivity. ADHD is frequent in children, and in up to 60% of the cases impairments persist into adulthood [1]. ADHD presents a high risk for developing co-morbid disorders, increasing the burden on social, educational, and professional aspects of life [2, 3]. Family and twin studies showed that ADHD is highly heritable, both in childhood and in adulthood, with heritability estimates range between 70 and 90% [4–6]. Despite this considerable heritability, the identification of risk genes has been challenging [3, 7], and one reason for this could be the genetic complexity of the disease. Identified candidate genes so far mainly belong to monoaminergic neurotransmitter pathways, especially dopaminergic and serotonergic signaling [8–12]. Different (hypothesis-free) approaches, including genome-wide linkage analyses and genome-wide association studies (GWASs), have been performed in order to detect additional genetic factors for ADHD. In line with the ‘common disease-common variant’ model, mostly common genetic factors have been investigated, which generally convey very small effect sizes [3]. However, GWASs of ADHD are only just reaching sufficiently large samples sizes to produce genome-wide significant results. Linkage analysis, a method useful for identification of genetic risk factors of larger effect size using family data, has also contributed to the identification of risk loci for ADHD. A meta-analysis of linkage studies in ADHD reported a significant region in the distal part of chromosome 16q [13]. Within this region, the *CDH13* gene was repeatedly found among the top-findings in GWASs [14]. In addition, linkage analysis in families from a genetic isolate in combination with association testing identified the *ADGRL3/LPHN3* gene as an ADHD risk factor [15, 16]. More evidence for involvement of less frequent genetic variants with potentially larger effect sizes comes e.g. from genome-wide studies of copy number variants (CNVs) [17–21] and initial exome-chip [22] and whole-exome sequencing (WES) work [23, 24]. In addition, WES has been successful in identifying rare risk alleles for other neurodevelopmental/psychiatric disorders, such as autism spectrum disorders (ASDs) and schizophrenia (e.g. [25, 26]).

In this study, we explored whether a combination of linkage analysis and WES in large multi-generational pedigrees is a viable approach to gene-finding in ADHD. We narrowed down the search area for rare variants by linkage analysis in three multi-generation pedigrees with multiple ADHD-affected members. Based on the WES applied to subsets of family members, we selected rare variants present in all (suggestive) linkage regions in each family. In line with the polygenic nature of ADHD, in which both common and rare genetic variants are likely to contribute to disease etiology, we subsequently used the extracted gene-sets to analyze the cumulative role of common and rare variants in persistent ADHD in an independent exome-chip data set (IMpACT consortium; *N* = 9365 [22]).

## Materials and methods

### Study participants

#### Multigenerational pedigrees

The study included three multi-generational families with multiple ADHD affected individuals (*N*_total_ = 70, *N*_ADHD_ = 41). The structure of the three families (Pedigree 1–3; P1–P3) is summarized in Table 1 and shown in Supplementary Figure 1. All families were of German origin and were ascertained through affected children referred to the outpatient clinic of the Department of Child and Adolescent Psychiatry and Psychotherapy, University Hospital Würzburg, Germany. For the index-child, strict inclusion and exclusion criteria were applied. Included index-children were aged ≥6 years and met criteria for ADHD combined subtype according to DSM-IV. Index-children had a birth weight >2000 g and Intelligence Quotient (IQ) > 80, did not show any neurological or severe somatic disorder, drug abuse or ASDs, and did not receive psychotropic medication (except for stimulant medication for ADHD). Detailed description of the diagnostic procedure for family members was reported previously [27]. The study was approved by the Ethics Committee of the Julius-Maximilians-University of Würzburg. Written informed consent was obtained from all participating individuals.

**Table 1:** Summary of the families included in this study

Family	Total	Affected	Unaffected	Unknown	WES	Genotyping
P1	11	9	1	1	5	7
P2	29	15	6	8	2	15
P3	30	17	8	5	3	15

*WES* whole-exome sequencing; genotyping indicates the number of family members with available genome-wide genotyping data for linkage analyses

#### Exome-chip data set

The data set, which did not include members of the families above, was genotyped on the Infinium Human CoreExome chip (Illumina, San Diego, CA, USA) and comprised 1846 adults with persistent ADHD and 7519 controls recruited from four different countries: Spain (615 cases and 932 controls), Norway (597 cases and 2598 controls), Germany (340 cases and 2 286 controls), and The Netherlands (294 cases and 1703 controls). Part of the Dutch controls were derived from the Nijmegen Biomedical Study (NBS, www.nijmegenbiomedischestudie.nl), a population-based survey conducted by the Departments of Epidemiology & Biostatistics and Clinical Chemistry of the Radboud University Medical Center [28]. Part of the Norwegian controls were derived from The Nord-Trøndelag Health Study (The HUNT study), a large population-based cohort [29]. Part of the German controls were derived from the Heinz–Nixdorf–Recall cohort, a large population-based cohort [30]. Persistent adult ADHD was diagnosed according to DSM-IV criteria. A detailed description of all samples and (genotyping) procedures was recently published [22], and a shortened version is included in the Supplementary Methods. ADHD cases were of European descent and were part of the International Multicenter persistent ADHD Collaboration (IMpACT [31]). The study was approved by the Ethics Committees of the respective universities and/or hospitals. All participants signed informed consent.

### Single-nucleotide polymorphism (SNP) genotyping and linkage analysis

Genome-wide SNP genotyping was performed on Affymetrix Genome-Wide Human SNP Array 6.0 (Affymetrix, Santa Clara, CA, USA). Microarray quality control parameters and genotype calls were generated with Affymetrix Genotyping Console v4.2.0.26 software (call rate > 0.99). Individuals were excluded if their call rate was below 97%. Genotyping data were filtered by removing SNPs with minor allele frequency (MAF) < 5%, missing genotypes > 5%, Mendelian errors > 10% for variants, or deviations from Hardy–Weinberg equilibrium (HWE, *P* ≤ 10^−6^). The remaining 665,362 SNPs were pruned to reduce linkage disequilibrium (LD) between markers using PLINK v1.07 software (http://pngu.mgh.harvard.edu/purcell/plink/ [32]) with pairwise *R*^2 > ^0.01 in sliding windows of 50 SNPs, moving in intervals of five SNPs. In total, 10,842 autosomal SNPs were included in the linkage analyses.

Multi-point linkage analyses of genome-wide SNP data were performed using Superlink Online SNP 1.1 [33], which can handle large pedigrees. Through parametric analysis, we computed logarithm of odds (LOD) scores using groups of markers with a window size of 10 markers. Based on the phenotypic characteristics of the pedigrees, a dominant model was specified with an allele frequency of 0.01 and a penetrance value of 0.99. The significance level was set at a LOD score of 3.3, but a score ≥2 was used to select candidate regions for further analyses (Supplementary Figure 2). The linked regions were bounded using 1 LOD-score intervals upstream and downstream from the marker with the highest LOD-score. Haplotypes of the regions of interest were constructed by using the haplotyping tool of Superlink Online SNP 1.1^33^.

### Whole-exome sequencing

For each family, two or more affected family members were strategically selected for WES based on meiotic distance and/or position in the pedigree (Table 1 and Supplementary Figure 1). Genomic DNA was extracted from blood samples according to standard protocols [27]. The exome was targeted by Agilent Sure Select Human All Exon 50 Mb Target Enrichment kit (Agilent Technologies, Santa Clara, CA, USA) and sequenced by single-end sequencing on the 5500xl SOLiD^TM^ System (Life Technologies, Carlsbad, CA, USA). High quality reads were mapped to the hg19 reference genome (UCSC genome browser) using the Lifescope 2.1 software (http://www.lifetechnologies.com/lifescope/) with default parameters. In addition, the SOLiD Lifescope Software v2.1 was used to call single-nucleotide variants (SNVs) using the diBayes algorithm. Variant annotation was done at the Department of Human Genetics of the Radboud university medical center using a pipeline developed in-house [34]. Variants were selected according to the following criteria: (i) variants with ≥20 reads, (ii) variants with a MAF < 1% in dbSNP (http://www.ncbi.nlm.nih.gov/SNP/), and (iii) variants present in all sequenced family members. Functional effects of variants were predicted by three different softwares: SIFT [35], PolyPhen-2 [36], and MutationTaster [37]. Conservation of variants was estimated by PhyloP [38] and Grantham score [39].

### Gene-based and gene-set analyses in the exome-chip data set

The cumulative effect of common and rare variants in selected genes and gene-sets was tested using the independent, recently published IMpACT exome-chip data set (Supplementary Methods; [22]). Statistical analyses were performed using the Multi-marker Analysis of GenoMic Annotation (MAGMA) software package (version 1.02; http://ctglab.nl/software/magma [40]) and the SNP-wide mean model for gene-based association analysis (for detailed description see Supplementary Methods). Importantly, all data sets (Spanish, Norwegian, German, and Dutch) were analyzed individually, followed by meta-analysis on the level of gene-based statistics. Fixed effects were used to perform meta-analysis by using the square root of the sample sizes as weights. An LD correction was applied, based on the estimates of the 1000 genome phase 1 European ancestry samples [41]. Data were analyzed following a step-wise approach (see also Supplementary Figure 2): first, an overall gene-set was created, including all genes identified by the approach described above (24 genes). We tested whether all genes together were jointly as a gene-set associated with persistent ADHD. Post hoc to the analysis of the overall gene-set, we also tested family-specific gene-sets to localize the effect. Subsequently, we performed a gene-based look-up of genes from family P2 (12 genes), and genes were considered gene-wide significant, if they reached the Bonferroni-corrected threshold adjusted for the number of genes within the gene-set (12 tests; *P* < 0.0042).

### Gene Ontology enrichment analysis

To assess whether the 12 prioritized genes from family P2 converge on biological shared functions, we tested for enrichment in Gene Ontology (GO) terms for biological processes using FUMA [42]. Overrepresentation of biological functions of prioritized genes was tested for by comparison with gene-sets obtained from the Molecular Signature Database (MsigDB) v5.2 (i.e., GO gene sets), using hypergeometric tests. The sets of background genes were derived from 19,264 protein-coding genes. Benjamini–Hochberg correction (FDR) was used for multiple test correction method for gene-set enrichment testing. Gene-set enrichments were considered significant at an adjusted *P*-value level < 0.05.

### Segregation analysis in family P2

To validate the presence of selected rare variants in the two sequenced individuals and to allow segregation analysis, all individuals of family P2 were genotyped for chr9:99404124G>C (rs151326868) and chr8:124346225T>C using PCR-based DNA sequencing. The locus of interest was amplified by conventional PCR and sequenced by direct Sanger sequencing (details and primer sequences are available upon request). Data obtained for the two variants were used to analyze the segregation with ADHD diagnosis.

## Results

### Linkage analysis across three families with ADHD

The main aim of the linkage analyses was to provide an additional filtering step for the WES data by narrowing down the genomic regions of interest. Linkage analysis was performed for each family individually, but also for all possible combinations of the three families. Informative individuals from each family were enrolled in the linkage study: nine individuals from family P1, 19 individuals from family P2, and 16 individuals from family P3 (Supplementary Figure 1). A total of 13 linkage regions with LOD-score ≥ 2 on chromosomes 6, 8, 9, 10, 11, 13, and 16 were identified (Supplementary Table 1, Supplementary Figures 3 and 4), and all were taken forward for subsequent analyses. Several significantly linked regions were observed by analyzing families together. The highest LOD scores (3.99 and 3.79) were located on chromosome 16 in the analysis combining P1 and P3 (Supplementary Table 1, Supplementary Figures 3 and 4). In family P1, six linkage regions were selected (LOD-score ≥ 2) for further analyses, but all of them needed the contribution of at least one additional family to reach significance. In family P2, nine linkage regions were identified, three of which specific to this family, and in family P3, nine linkage regions were identified of which five were specific to this single family (all linkage regions with LOD-score ≥ 2; Table 2 and Supplementary Table 1).

**Table 2:** List of candidate regions and genes selected based on the linkage analysis in each family

			Gene-set analysis	
Family	LR selected	Genes with rare variants in WES	*P*_self-contained_	*P*_competitive_
P1	8:118608158–124649389	-----	0.2838	0.4512
	9:7754113–15568230	*TYRP1*, *FREM1*		
	9:97466973–102213749	-----		
	11:115218677–120365028	*NXPE1*,*TMEM25*, *HYOU1*, *VPS11*, *ABCG4, CCDC153*		
	16:63079319–66386711	*HSF4*		
	16:81159781–83154022	*DYNLRB2*, *PKD1L2*, *PLCG2*, *OSGIN1, MBTPS1*		
P2	8:118608158–124649389	*DEPTOR*, *ATAD2*	0.0066	0.0042
	9:7754113–15568230	*PTPRD*, *TYRP1, FREM1*		
	9:97466973–102213749	*HSD17B3*, *AAED1*, *ANP32B*^a^, *TBC1D2*		
	10:56177098–58789387	*PCDH15*		
	10:64668048–65875491	-----		
	11:21968768–29134515	*ANO3*		
	11:115218677–120365028	*BUD13*, *VPS11*		
	13:106701406–109091885	-----		
	16:63079319–66386711	-----		
P3	6:203878–460901	-----	0.1368	0.1393
	6:3446942–4470581	-----		
	9:97466973–102213749	-----		
	10:14311273–15844850	-----		
	10:64668048–65875491	-----		
	11:115218677–120365028	*BUD13*, *TMEM25, VPS11*		
	13:106701406–109091885	-----		
	16:63079319–66386711	*CDH5*		
	16:81159781–83154022	*MBTPS1*		

Genes were included if they were present in the linkage region (LR; ± 1 Mb) with LOD ≥ 2, to which the family was contributing and if they harbored a rare variant (according to our selection criteria).

^a^No variants were observed in the *ANP32B* gene in IMpACT exome-chip data. Gene-set-based association analysis used meta-analytic exome-chip data from 9365 individuals (1846 ADHD patients and 7519 controls [22]).

### Whole-exome sequencing analysis

A total of ten ADHD-affected family members were included in WES: five from family P1 (ID1, ID2, ID4, ID5, and ID11), two from family P2 (ID21 and ID26), and three from family P3 (ID17, ID19, and ID20)) (Supplementary Figure 1). We obtained an average of 5.46 billion bases of sequence per individual and about 82.2% (~4.49 billion bp) of the total bases mapped to the exomes, with a mean of 85-times coverage (for WES sequencing statistics per individual see Supplementary Table 1). Based on our selection criteria, the average number of shared rare variants present in each family was 1235 across the exome. Applying filtering based on the identified linkage regions, a total of 20 variants were selected from family P1, 13 variants from family P2, and five variants from family P3 (Supplementary Table 3).

### Association analyses and candidate gene identification in an independent sample

All genes within linkage regions containing at least one of the selected rare variants were included in a list of candidate genes (Table 2). Gene-set analysis was performed based on this list using exome-chip data from an independent sample of 1846 adults with persistent ADHD and 7519 controls [22]. Following testing of the overall gene-set (24 genes), we also tested gene-sets resulted from each family separately (Table 2). Meta-analysis of the individual exome-chip samples showed significant association of the overall gene-set in both self-contained and competitive tests (*P*_self-contained_ = 0.0063 and *P*_competitive_ = 0.0103, Table 2). The significant effect of the general gene-set was mainly driven by the effect of genes selected based on the linkage analyses in family P2 (*P*_self-contained_ = 0.0066 and *P*_competitive_ = 0.0042, 12 genes, Table 2), with additional minor contributions of the gene-sets resulting from the linkage analysis in the other two families (Supplementary Table 4). Focusing on individual genes of the P2 gene-set, gene-based analysis revealed that the *AAED1* gene was significantly associated with persistent ADHD (*P* = 0.0039). Another gene in this gene set—*ATAD2*—yielded suggestive significance after correction for multiple testing (*P* = 0.0072, Table 3). For both genes, association was driven entirely by rare variants (Supplementary Tables 5 and 6).

**Table 3:** Gene-based association results for the family P2 gene-set using IMpACT exome-chip data of 9365 individuals (1846 ADHD patients and 7519 controls; [22])

Gene	*N* variants	*P*
*AAED1*	5	0.0039^a^
*ATAD2*	5	0.0072
*BUD13*	11	0.0136
*ANO3*	9	0.1308
*DEPTOR*	6	0.2279
*TYRP1*	8	0.2824
*TBC1D2*	12	0.3181
*VPS11*	9	0.3258
*PCDH15*	27	0.3434
*PTPRD*	28	0.6350
*HSD17B3*	5	0.7097
*FREM1*	23	0.9460

^a^Significant association after Bonferroni correction (12 tests, *P* < 0.00417).

To assess whether the 12 prioritized genes of the family P2 gene-set converged on biological functions or pathways, we tested for enrichment in GO terms (biological processes). Four significantly enriched GO-terms were detected, including ‘regulation of vesicle fusion’ (*P*_adjusted_ = 0.0166) and ‘cell–cell adhesion via plasma membrane adhesion molecules’ (*P*_adjusted_ = 0.0328) (Supplementary Figure 5).

### Single variant validation and familial segregation analysis

Going back to the WES data of family P2, one rare missense variant was identified in both candidate genes (*AAED1* and *ATAD2*) from the gene-based analysis. The variant rs151326868, located in *AAED1* (chr9:99404124G>C), was predicted to be deleterious in all pathogenicity tests (Polyphen2, SIFT, and MutationTaster), was highly conserved (PhyloP>2.7 and Grantham score >80; Supplementary Table 7), and rare (MAF = 4.38 × 10^−4^ in the ExAC browser). The SNV chr8:124346225T>C in *ATAD2* was predicted to be deleterious only by MutationTaster, showed low conservation scores, and had very low MAF in the ExAC browser (8.24 × 10^−06^; Supplementary Table 7). The variant rs151326868 in *AAED1* was also present in the exome-chip data (exome-chip marker exm764638; Supplementary Tables 5 and 6), the SNV chr8:124346225T>C in *ATAD2* was not.

**Fig. 1:**
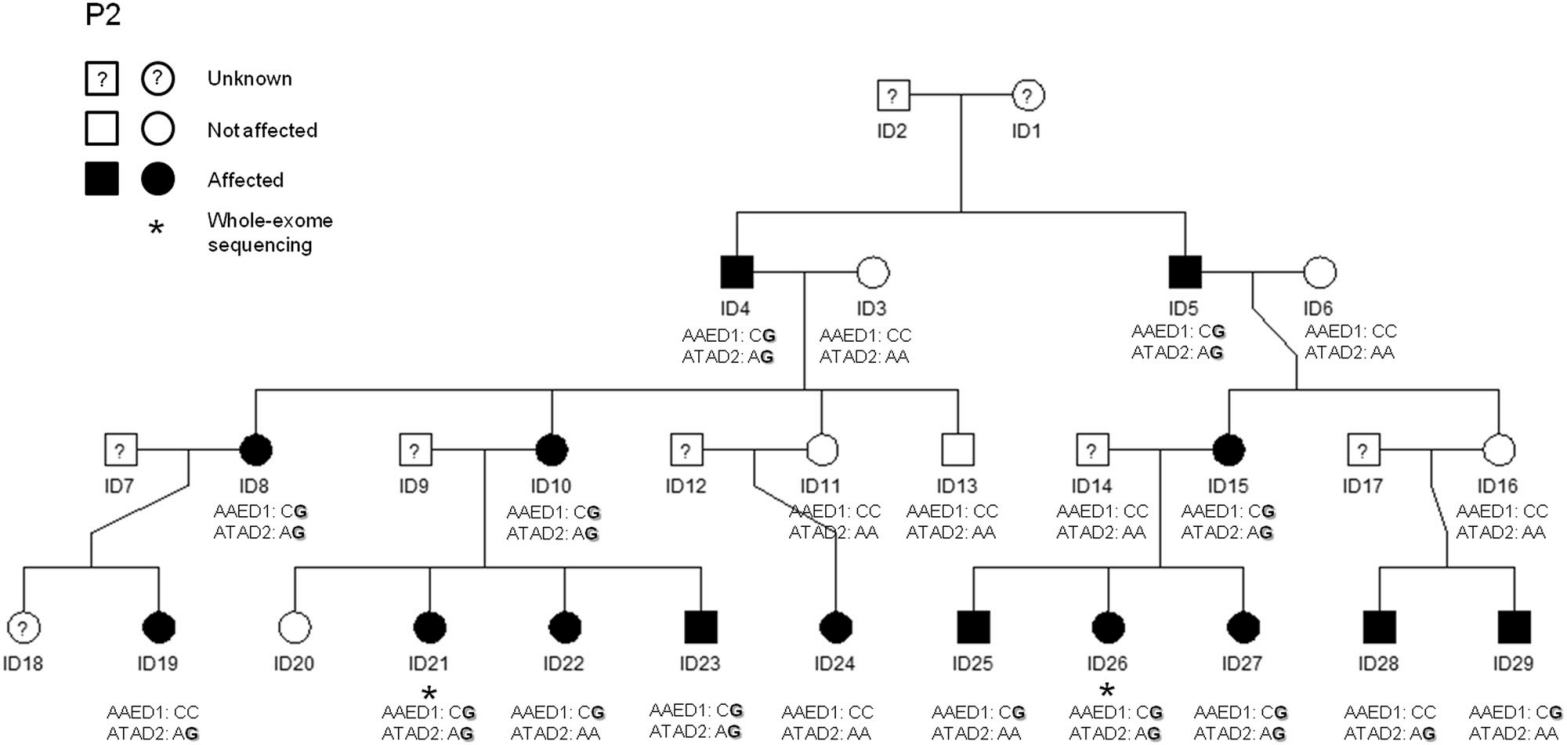
Segregation analysis for rs151326868 (chr9:99404124G>C; *AAED1*gene) and the SNV at chr8:124346225T>C (*ATAD2*gene) in family P2. ADHD-affected individuals are depicted by black symbols, unaffected family members are shown by white symbols and individuals with unknown ADHD status are represented by a question mark in the symbol. An asterisk beneath an individual indicates that DNA was used for whole-exome sequencing analysis. Non-reference alleles are depicted in bold.

Sanger sequencing of these two rare variants in *AAED1* and *ATAD2* in all members of family P2 for whom DNA was available confirmed the presence of these variants in the two sequenced individuals and allowed segregation analysis. None of the healthy individuals carried either of the variants, 93% of the affected individuals (14/15) carried at least one of the two variants, and 60% of the affected individuals (9/15) carried both variants (Fig. 1).

## Discussion

In this study, we aimed to identify novel genetic risk factors for ADHD by combining linkage analyses and WES in multi-generation families. We predicted that through a family-based approach, we would be able to limit genetic heterogeneity, since rare variants with potentially higher penetrance may cause the disorder in familial ADHD cases. Linkage analyses revealed four genomic regions with a LOD score ≥3.3 and 15 additional regions with a LOD score ≥2. Within these regions, we identified 38 rare variants within 25 genes across the three families. All genes together, and particularly the gene-set from family P2, were significantly associated with persistent ADHD in the independent exome-chip sample. Moreover, the *AAED1* gene reached gene-wide significance in that sample, and a rare variant in *AAED1* (rs151326868) segregated with ADHD in family P2.

Different designs can be used for WES studies aiming at identifying rare variants linked to complex diseases. In schizophrenia [26] and ASDs [43–45], large sample sizes of cases and controls have been used to find genes implicated in disease through rare variants in multiple patients. For ADHD, data sets have not yet reached the critical size to allow for a genome-wide, hypothesis-free analysis of WES data, but an initial study analyzed a pre-defined gene-set of interest and found evidence for enrichment of rare variants in cases [23]. A second approach, that has successfully been applied in other neurodevelopmental disorders, especially in ASDs [44, 46], is a trio-approach in sporadic patients and their parents. In this approach, one is assuming that the occurrence of the disorder in the patient is due to a de novo mutation. In ADHD, this design may seem less promising since the disorder does not reduce reproductive fitness as it does in ASDs [47] and, therefore, sporadic cases are less frequently described and familial aggregation of ADHD is frequently observed [48]. However, recent evidence from Swedish population registries suggests that ADHD risk is strongly increased in the offspring of fathers older than 45 years [49], which seems to be linked to an age-related increasing mutation rate in the paternal germline [50]. Our own work also suggests that the cognitive profile of families with only one affected individual differs from that of families with more cases [51, 52], which may suggest that the trio-design could also be successful in ADHD. The third design, which we employed in the current study, is the extended pedigree-based approach, in which one screens for segregation of rare variants with disease across multiple affected individuals. Knowledge on the etiology of ADHD is, however, still limited, and therefore, ranking and prioritization of potential candidate genes is challenging. With this in mind, our combined linkage and WES approach did help to efficiently limit the list of potentially causative variants in a data-driven way. Filtering WES variants by linkage analysis has earlier been shown to be an effective tool for prioritizing common and exome variants in extended families with ADHD [14] or ASD [53].

We extended the family-based approach by testing the effects of observed genes carrying rare variants in an independent, large sample of exome-chip data. Importantly, most of the selected rare variants in the genes included in the gene-set analysis of family P2 are exonic and non-synonymous variants, so the overall result in the case–control analyses would not be affected by more stringent selection criteria for rare variants that are frequently used in WES studies (e.g., being functionally relevant). Specifically, the significant gene-based association of *AAED1* would remain. Utilizing this independent sample, we showed that (some of the) identified genes may be relevant to ADHD in the population, thereby generalizing the findings from the single family. This approach also enabled us to study the cumulative effect of rare and common genetic variants in the identified candidate genes for association with persistent ADHD, maximizing power to find association by taking into account allelic heterogeneity [8, 22].

Importantly, our work supports the notion that—despite the apparent dominant segregation pattern - ADHD is not a monogenic disorder in the pedigrees investigated. Linkage analyses revealed several (suggestive) signals per family, suggesting that several genes/loci may carry risk variants for ADHD in each of those. Based on the linkage analyses, we did not expect a single gene or single locus to be associated with the clinical phenotype nor perfect co-segregation pattern of the rare, non-reference allele with ADHD in subsequent segregation analyses. A main contributing factor to the observed patterns may be assortative mating, which is common in ADHD [54] (and e.g. present in family P1). Although we were quite liberal in selecting regions for further analysis (through including suggestive linkage signals), the observed pattern is similar to findings in previous linkage studies of ADHD [13, 14] and other neurodevelopmental disorders (e.g., for ASDs [53]).

The prioritized genes in the gene-set of family P2 converged on the biological function of vesicle fusion, which adds to the relevance of our findings, since the process of vesicle fusion to plasma (e.g., synaptic) membrane is closely related to the mechanism of neurotransmitter release. The *AAED1* gene (coding for the AhpC/TSA Antioxidant Enzyme Domain Containing 1 protein) was significantly associated with persistent ADHD in the exome-chip sample, and the rare variant in this gene (rs151326868; MAF for C-allele in ExAC = 4.38 × 10^−4^) segregated with ADHD risk in family P2. AAED1 strongly binds and interacts with the Protein Kinase C-Alpha-Binding Protein (PICK1) [55]. PICK1 binds to the dopamine transporter (DAT), more specifically to its carboxyl terminus, and is an important regulator of DAT trafficking in presynaptic sites of dopaminergic neurons [56]. Additionally, a direct and functional interaction between PICK1 and dopamine D_3_ receptors (D_3_R) has been reported [57]. Furthermore, PICK1 has a role in glutamate receptor regulation [58], and a recent study revealed that a glutamate gene-set showed association with the severity of hyperactivity/impulsivity in an ADHD case-only sample [59]. In addition, adult *Pick1* knockout mice show several behavioral abnormalities, such as hyperactivity and electrophysiological deficits in the prefrontal cortex [60]. With the prominent involvement of dopamine regulation in ADHD, as e.g. the dopaminergic system plays an important role in planning and initiation of motor responses, activation, switching, reaction to novelty, and reward processing [3], these molecular findings suggest a link between genetic variation in *AAED1*, dopaminergic and glutamatergic signaling, and ADHD risk. Thus, studies of the *AAED1* variant’s functional impact in carrier-derived neurons of dopaminergic and glutamatergic specification, which have been differentiated from induced pluripotent stem cells (iPSCs), are currently being conducted (E. Svirin & K.P. Lesch, unpublished results).

Our combined approach of linkage and WES also identified a rare genetic variant in the *ATAD2* gene, coding for the ATPase family AAA domain-containing protein 2, and gene-based analysis of this gene revealed suggestive association with persistent ADHD. However, a neuronal function of this gene has not been described yet.

The findings described here need to be interpreted in light of several strengths and limitations. Although we considered only three families, we identified *AAED1* as a novel ADHD candidate gene, showing that combining linkage analysis and WES can be an efficient strategy to prioritize ADHD-associated genes/variants. In contrast to previous studies focusing on pre-defined gene-sets [23, 61], we performed an exome-wide search for rare variants. Additionally, we validated the association of the newly identified ADHD risk genes in an independent sample. However, two main types of genetic variation, which may have helped us to find contributing genes in families P1 and P3, remained unstudied. Firstly, genetic variation located in intronic and intergenic regions may be discovered by using whole-genome sequencing approaches. Alternatively, common variants in regulatory regions close to the genes of interest may be imputed and then included in association analyses. Since we know from studies in other psychiatric disorders that many risk variants are located within regulatory regions [62], genetic variation in those regions probably also contributes to the genetic architecture of ADHD. Secondly, CNVs could play a role in the etiology of ADHD, since prior studies have noted an enrichment of large CNVs in ADHD cases [17, 20, 63, 64], particularly in genes related to neurodevelopment [21]. Moreover, future studies may aim to integrate data from both rare variants and the common polygenic load in those families, in order to obtain a more complete picture on the genetic architecture of ADHD in the individual families.

In conclusion, we provide evidence for the role of rare variants in protein-coding genes in the etiology of ADHD. Our data adds to the notion that less frequent variants provide an additional source of relevant genetic risk factors, which received little attention in ADHD genetics so far. Moreover, we show that genes harboring rare genetic variants in individual families are associated with persistent ADHD in an independent sample. Therefore, this study suggests that the combination of linkage analyses and WES provides a practical approach for gene identification in genetically complex neurodevelopmental disorders, such as ADHD.

## Electronic supplementary material

Supplementary Methods

Supplementary Figure 1

Supplementary Figure 2

Supplementary Figure 5

Supplementary Figure 3

Supplementary Figure 4

Supplementary Table 5

Supplementary Table 6
