# Supplementary material for: Identification of ADHD risk genes in extended pedigrees by combining linkage analysis and whole-exome sequencing
Source: Mol Psychiatry. 2018 Aug 16;25(9):2047–57. doi: 10.1038/s41380-018-0210-6 (PMC7473839; doi:10.1038/s41380-018-0210-6)
Supplement: Supplementary file 1 — Supplementary Methods [file 41380_2018_210_MOESM1_ESM.docx]

**SUPPLEMENTARY INFORMATION**

**Identification of ADHD risk genes in extended pedigrees by combining linkage analysis and whole-exome sequencing**

Jordi Corominas, Marieke Klein, Tetyana Zayats, Olga Rivero, Georg C. Ziegler, Marc Pauper, Kornelia Neveling, Geert Poelmans, Charline Jansch, Evgeniy Svirin, Julia Geissler, Heike Weber, Andreas Reif, Alejandro Arias Vasquez, Tessel E. Galesloot, Lambertus A.L.M. Kiemeney, Jan K. Buitelaar, Josep-Antoni Ramos-Quiroga, Bru Cormand, Marta Ribasés, Kristian Hveem, Maiken Elvestad Gabrielsen, Per Hoffmann, Sven Cichon, Jan Haavik, Stefan Johansson, Christian P. Jacob, Marcel Romanos, Barbara Franke, Klaus-Peter Lesch

The supplementary information contains:

- Supplementary methods
- Supplementary tables 1-7
- Figure legends for Figure 1 and Supplementary figures 1-5

**SUPPLEMENTARY METHODS**

*Exome-chip sample*

*Subjects*

The exome-chip sample has been described previously^1^. All adult ADHD patients examined in the original study^1^ were volunteers enrolled through the International Multicenter persistent ADHD CollaboraTion (IMpACT; http://www.impactadhdgenomics.com/). All patients were diagnosed with ADHD according to DSM-IV criteria, i.e. ADHD symptoms were present before 7 years of age. Controls were recruited either at an IMpACT site (Germany, The Netherlands, UiB Norway, and Spain) or through population studies (Germany, The Netherlands, and Norway [The Nord-Trøndelag Health Study; The HUNT study]). All subjects were of European descent which was proven by means of self-report, together with multi-dimensional scaling (MDS; reported in the original publication;^1^). All participants provided signed informed consent in accordance with the Declaration of Helsinki. The study was approved by ethics committees in each collaborating country at the corresponding recruitment center (for details see^1^). Discovery samples were available from four European IMpACT sites: Germany, The Netherlands, Norway, and Spain. Additional Dutch controls for the discovery analyses were derived from the Nijmegen Biomedical Study (NBS, www.nijmegenbiomedischestudie.nl), a population-based survey conducted by the Departments of Epidemiology & Biostatistics and Clinical Chemistry of the Radboud University Medical Center^2^. Approval to conduct the study was obtained from the Institutional Review Board. Controls were unselected for ADHD symptoms. A detailed description of all samples is provided in the Supplementary Appendix 1 of the original publication^1^. The replication sample was not utilized in this study. The gene-sets were analyzed in the discovery sample only.

*Genotyping, genotype calling and quality control*

All subjects of the discovery stage were genotyped on the InfiniumHumanExome array (Illumina, San Diego, CA, USA). The German cases, the full Dutch sample, and the full Spanish sample were genotyped on HumanExome-12v1-1_A; the German controls and the HUNT Norwegian sample of controls were genotyped on HumanExome-12v1_A; and the UiB Norwegian sample was genotyped on HumanExome-12v1_B version of the chip. All genotypes were processed using Illumina GenomeStudio V2011.1 software, with additional genotype assignments implemented in zCall^3^. Best practice guidelines were used to perform quality control (QC) of genotype calls in GenomeStudio^4^. Further QC was carried out on all data sets using PLINK^5^, with the following steps: (1) genotyping rate threshold was set to 98% both for individuals and for SNVs, (2) Hardy–Weinberg test threshold was set to P<1.00x10^−05^, (3) overall heterozygosity of individuals was screened based on common (minor allele frequency (MAF)⩾1%) and rare (MAF<1%) SNVs separately, with outliers defined as those outside the range of mean ± 3SD of the total heterozygosity observed in a sample, (4) relatedness (PI_HAT) threshold was set to 10% and (5) ethnic homogeneity was guaranteed by means of MDS with HapMap3 populations. Genotype calling and all QC steps were performed for each data set individually. Those samples that were collected in the same country (namely German cases and German controls, Dutch cases and Dutch controls, Norwegian UiB and HUNT samples) were merged using PLINK and additional QC steps were implemented. Specifically, the screening for heterozygosity, cryptic relatedness, and population outliers was performed once more as described above. Finally, four data sets were produced: a combined German sample, a combined Dutch sample, a combined Norwegian sample, and a Spanish sample.

*Statistical analyses*

Statistical analyses were divided into two main stages: (1) examination of rare coding SNVs (MAF < 1%) and (2) examination of common SNVs (MAF ≥ 1%) with replication in and independent sample.

Prior to the analyses, each subject’s genetic substructure characteristics were estimated with principal components analysis implemented in EIGENSTRAT software for each data set individually^6^. Calculation of principal components was performed based only on individuals and SNVs revealing high genotyping rate (⩾99%) and common variants (MAF⩾ 1%) after removal of strand ambiguous SNVs and those in high LD (r2<0.2). Long stretches of LD were also removed prior to calculation of principal components^7^. Rare variants were defined as those with MAF < 1%. The variants were combined per gene and tested for association with adult ADHD in RAREMETAL^8^. Common SNVs were defined as those with MAF ≥ 1%. The variants were tested using PLINK^5^, assuming an additive model.

*Gene-based and gene-set analyses in the exome-chip dataset*

Statistical analyses were performed using the Multi-marker Analysis of GenoMic Annotation (MAGMA) software package (version 1.02; <http://ctglab.nl/software/magma>^9^). The subjects’ genetic substructure was taken into account by including principal components, reflecting their genetic characteristics, as covariates^1^. Genome-wide SNP data from a reference panel (1000 Genomes, v3 phase1^10^) was annotated to NCBI Build 37.3 gene locations. This gene annotation file was used to map the exome chip data from the different samples to assign genetic variants to genes, followed by the calculation of gene-based p-values for each sample. For this, a degree of association was calculated for each gene based on both common and rare variants by using principal component regression. Rare variants were defined as those with MAF < 1%, and a burden score was generated for each gene, computed as weighted sum of all rare variants in that gene. Burden scores are implemented as a pre-processing step. A threshold is first specified on either MAF or MAC to designate SNPs as ‘rare’. For each gene, one or more burden scores are then created by summing the rare variants in that gene. The individual rare variants are then removed from the gene and replaced by these burden scores. Analysis of that gene then proceeds as normal, using the chosen gene analysis model (specified by --gene-model) to analyze the burden scores and remaining common variants (if any). No variants in *ANP32B* were observed in the IMpACT exome-chip data and, therefore, only 24 genes were analyzed. For gene-based analyses, single variant p-values within a gene were transformed into a gene-statistic by taking the mean of the χ2-statistic among variants in each gene. To account for LD, the 1000 Genomes Project European sample was used as a reference to estimate the LD between variants within (the vicinity of) genes (<http://ctglab.nl/software/MAGMA/ref_data/g1000_ceu.zip>). Gene-wide p-values were converted to z-values reflecting the strength of the association of each gene with ADHD risk, with higher z-values corresponding to stronger associations. Subsequently, we tested whether all 24 genes in the overall gene-set were jointly associated with persistent ADHD, using an intercept-only linear regression model including a subvector corresponding to the genes in the gene-set. This self-contained analysis evaluates whether the regression coefficient of this regression was ≥0, testing whether the overall gene-set showed association with persistent ADHD. Next, we tested whether genes in this gene-set were more strongly associated with persistent ADHD than others genes in the genome. With this competitive test, differences between the association of the gene-set to genes outside this gene-set is tested, accounting for the polygenic nature of a complex disorder like ADHD. Potential confounders, such as gene size and gene density, were included in the competitive test. Because of this, we were more interested in the competitive than the self-contained test for the current analyses.

**REFERENCES**

1 Zayats T, Jacobsen KK, Kleppe R, Jacob CP, Kittel-Schneider S, Ribases M et al. Exome chip analyses in adult attention deficit hyperactivity disorder. Transl Psychiatry 2016; 6: e923.

2 Galesloot TE, Vermeulen SH, Swinkels DW, de Vegt F, Franke B, den Heijer M et al. Cohort Profile: The Nijmegen Biomedical Study (NBS). Int J Epidemiol 2017.

3 Goldstein JI, Crenshaw A, Carey J, Grant GB, Maguire J, Fromer M et al. zCall: a rare variant caller for array-based genotyping: genetics and population analysis. Bioinformatics 2012; 28: 2543-2545.

4 Grove ML, Yu B, Cochran BJ, Haritunians T, Bis JC, Taylor KD et al. Best practices and joint calling of the HumanExome BeadChip: the CHARGE Consortium. PLoS One 2013; 8: e68095.

5 Purcell S, Neale B, Todd-Brown K, Thomas L, Ferreira MA, Bender D et al. PLINK: a tool set for whole-genome association and population-based linkage analyses. Am J Hum Genet 2007; 81: 559-575.

6 Price AL, Patterson NJ, Plenge RM, Weinblatt ME, Shadick NA & Reich D. Principal components analysis corrects for stratification in genome-wide association studies. Nat Genet 2006; 38: 904-909.

7 Price AL, Weale ME, Patterson N, Myers SR, Need AC, Shianna KV et al. Long-range LD can confound genome scans in admixed populations. Am J Hum Genet 2008; 83: 132-135; author reply 135-139.

8 Feng S, Liu D, Zhan X, Wing MK & Abecasis GR. RAREMETAL: fast and powerful meta-analysis for rare variants. Bioinformatics 2014; 30: 2828-2829.

9 de Leeuw CA, Mooij JM, Heskes T & Posthuma D. MAGMA: generalized gene-set analysis of GWAS data. PLoS Comput Biol 2015; 11: e1004219.

10 Genomes Project C, Abecasis GR, Altshuler D, Auton A, Brooks LD, Durbin RM et al. A map of human genome variation from population-scale sequencing. Nature 2010; 467: 1061-1073.

11 Watanabe K, Taskesen E, van Bochoven A & Posthuma D. FUMA: Functional mapping and annotation of genetic associations. bioRxiv 2017.

**SUPPLEMENTARY TABLES**

**Supplementary Table 1.** Candidate linkage regions selected for further analysis containing a maximum logarithm of the odds (LOD) score higher than 2 in at least one of analysis performed.

| **Chr** | **Start marker** | **End marker** | **Region** | **Max. LOD score** | **Analysis** |
| --- | --- | --- | --- | --- | --- |
| 6 | rs9392298 | rs1113387 | 203878-460901 | 3.003 | Pedigree 3 |
| 6 | rs6596970 | rs12211028 | 3446942-4470581 | 3.003 | Pedigree 3 |
| 8 | rs1993979 | rs11776334 | 118608158-124649389 | 2.2030 | Pedigree 1 and 2 combined |
| 9 | rs10758897 | rs7875367 | 7754113-15568230 | 3.6246 | Pedigree 1 and 2 combined |
| 9 | rs7847004 | rs7847405 | 97466973-102213749 | 2.0735 | All pedigrees combined |
| 10 | rs10906640 | rs17395983 | 14311273-15844850 | 2.3948 | Pedigree 3 |
| 10 | rs1612430 | rs7919353 | 56177098-58789387 | 2.5803 | Pedigree 2 |
| 10 | rs10509177 | rs2601705 | 64668048-65875491 | 2.713 | Pedigree 2 and 3 combined |
| 11 | rs324256 | rs4923591 | 21968768-29134515 | 2.0918 | Pedigree 2 |
| 11 | rs4938200 | rs12364480 | 115218677-120365028 | 3.3631 | Pedigree 1 and 3 combined |
| 13 | rs9558670 | rs16972472 | 106701406-109091885 | 2.5168 | Pedigree 2 and 3 combined |
| 16 | rs153331 | rs7198762 | 63079319-66386711 | 3.9898 | Pedigree 1 and 3 combined |
| 16 | rs7200569 | rs11150541 | 81159781-83154022 | 3.7949 | Pedigree 1 and 3 combined |

**Supplementary Table 2.** WES sequencing statistics per individual.

| **Family** | **ID** | **bp sequenced** | **bp mapped on-target** | **Total number reads** | **Total mapped reads on-target** | **Percentage of**  **on-target reads (%)** | **Mean Coverage** |
| --- | --- | --- | --- | --- | --- | --- | --- |
| 1 | 1 | 4 432 495 337 | 3 900 102 767 | 91 460 024 | 79 681 085 | 87.12 | 65.08 |
| 1 | 2 | 5 012 989 993 | 4 411 768 262 | 103 829 553 | 90 405 567 | 87.07 | 73.31 |
| 1 | 4 | 4 185 095 550 | 3 543 760 472 | 86 630 343 | 72 553 451 | 83.75 | 57.42 |
| 1 | 5 | 4 769 484 984 | 4 217 957 342 | 99 420 668 | 87 006 501 | 87.51 | 71.26 |
| 1 | 11 | 4 453 320 723 | 3 911 917 639 | 92 094 198 | 80 066 716 | 86.94 | 64.52 |
| 2 | 21 | 6 455 493 150 | 5 851 516 254 | 135 431 851 | 121 571 589 | 89.77 | 106.42 |
| 2 | 26 | 7 308 540 150 | 6 672 649 219 | 153 224 671 | 138 653 596 | 90.49 | 121.56 |
| 3 | 17 | 6 270 779 135 | 5 667 764 511 | 133 028 891 | 118 744 178 | 89.26 | 103.01 |
| 3 | 19 | 4 931 660 430 | 4 303 489 528 | 101 845 716 | 87 873 677 | 86.28 | 72.42 |
| 3 | 20 | 6 818 067 496 | 6 191 537 997 | 144 434 109 | 129 623 720 | 89.75 | 112.32 |

**Supplementary Table 3.** List of rare variants identified using WES within linkage regions. In total, we identified 38 rare variants, of which 32 were unique and located within a gene.

| **Family** | **Variant ID** | **rs ID** | **Gene name** | **Gene component** | **Type** |
| --- | --- | --- | --- | --- | --- |
| 1 | chr9:12708958->A | ------ | *TYRP1* | Splice site | ------- |
| 1 | chr9:14775859G>A | rs10733289 | *FREM1* | Exon | Synonymous |
| 1 | chr11:114421857T>C | rs78453693 | *NXPE1* | Intron | ------ |
| 1 | chr11:114421861G>A | rs76118887 | *NXPE1* | Intron | ------ |
| 1 | chr11:114421895G>A | rs78257939 | *NXPE1* | Intron | ------ |
| 1 | chr11:114421927G>A | rs78842965 | *NXPE1* | Intron | ------ |
| 1 | chr11:114421962G>A | rs75409523 | *NXPE1* | Intron | ------ |
| 1 | chr11:118405343G>A | rs512849 | *TMEM25* | Intron | ------ |
| 1 | chr11:118927013->C | rs75923954 | *HYOU1* | Intron | ------ |
| 1 | chr11:118939939->C | rs199535207 | *VPS11* | Exon | Non-synonymous |
| 1 | chr11:119031841->C | ------ | *ABCG4* | UTR | ------ |
| 1 | chr11:119065484->C | ------ | *CCDC153* | Intron | ------ |
| 1 | chr16:67197776->G | ------ | *HSF4* | Intron | ------ |
| 1 | chr16:80581631G>A | rs6564764 | *DYNLRB2* | Intron | ------ |
| 1 | chr16:81145675T>C | rs8059153 | *PKD1L2* | Intron | ------ |
| 1 | chr16:81242198G>A | rs7499011 | *PKD1L2* | Exon | Non-synonymous |
| 1 | chr16:81816787T>A | rs4476171 | *PLCG2* | Intron | ------ |
| 1 | chr16:83992785G>A | rs2665296 | *OSGIN1* | Intron | ------ |
| 1 | chr16:84132628->AC | rs202131529 | *MBTPS1* | Intron | ------ |
| 2 | chr8:120940874T>C | rs62528677 | *DEPTOR* | Intron | ------ |
| 2 | chr8:124346225T>C | ------ | *ATAD2* | Exon | Non-synonymous |
| 2 | chr9:9425588->ACTT | rs200022326 | *PTPRD* | Intron | ------ |
| 2 | chr9:12694274G>A | rs61752937 | *TYRP1* | Exon | Non-synonymous |
| 2 | chr9:14775859G>A | rs10733289 | *FREM1* | Exon | Synonymous |
| 2 | chr9:99064202->A | ------ | *HSD17B3* | Intron | ------ |
| 2 | chr9:99404124G>C | rs151326868 | *AAED1* | Exon | Non-synonymous |
| 2 | chr9:100756891->T | ------ | *ANP32B* | Intron | ------ |
| 2 | chr9:100995758G>T | rs879368 | *TBC1D2* | Exon | Non-synonymous |
| 2 | chr10:55590946CAGAC>T | ------ | *PCDH15* | Intron | ------ |
| 2 | chr11:26574783G>- | rs143835466 | *ANO3* | Splice site | ------ |
| 2 | chr11:116633913A>G | ------ | *BUD13* | Exon | Non-synonymous |
| 2 | chr11:118939939->C | rs199535207 | *VPS11* | Exon | Non-synonymous |
| 3 | chr11:116633913A>G | ------ | *BUD13* | Exon | Non-synonymous |
| 3 | chr11:118405343G>A | rs512849 | *TMEM25* | Intron | ------ |
| 3 | chr11:118939939->C | rs199535207 | *VPS11* | Exon | Non-synonymous |
| 3 | chr16:66432304ACCACCCC>- | rs113303884 | *CDH5* | Intron | ------ |
| 3 | chr16:84132628->AC | rs202131529 | *MBTPS1* | Intron | ------ |

**Supplementary Table 4.** Results of the gene-set association analyses of the individual IMpACT exome-chip samples ^1^.

| **Cohort** | **N (cases/controls)** | **Gene-set** | **N genes** | **P_self-contained_** | **P_competitive_** |
| --- | --- | --- | --- | --- | --- |
| **Spain** | (615/932) | General | 23 | 0.074232 | 0.034095 |
|  |  | P1 | 13* | 0.8862 | 0.8772 |
|  |  | P2 | 12 | 0.00067007 | 0.00013433 |
|  |  | P3 | 5 | 0.10719 | 0.026187 |
| **Norway** | (597/2 598) | General | 23 | 0.038002 | 0.055531 |
|  |  | P1 | 13* | 0.038847 | 0.05538 |
|  |  | P2 | 12 | 0.051919 | 0.078343 |
|  |  | P3 | 5 | 0.67716 | 0.65549 |
| **Germany** | (340/2 286) | General | 24 | 0.21395 | 0.30365 |
|  |  | P1 | 14 | 0.38634 | 0.50658 |
|  |  | P2 | 12 | 0.50793 | 0.53963 |
|  |  | P3 | 5 | 0.074312 | 0.18876 |
| **Netherlands** | (294/1 703) | General | 24 | 0.16794 | 0.11289 |
|  |  | P1 | 14 | 0.63434 | 0.66457 |
|  |  | P2 | 12 | 0.30359 | 0.16463 |
|  |  | P3 | 5 | 0.41871 | 0.41045 |

*No variants were observed in the *DYNLRB2* gene in this sample of the IMpACT exome-chip data.

1 Zayats T, Jacobsen KK, Kleppe R, Jacob CP, Kittel-Schneider S, Ribases M et al. Exome chip analyses in adult attention deficit hyperactivity disorder. Translational psychiatry 2016; 6: e923.

**Supplementary Table 5.** Minor allele frequencies and summary statistics for common variants from meta-analysis of the exome-chip data.

>>> see file *Supplementary_Table_S5.xlsx* <<<

**Supplementary Table 6.** Count data for all markers of the exome-chip data is shown per site.

>>> see file *Supplementary_table_S6_counts_only.xlsx* <<<

**Supplementary Table 7.** Information on two rare variants from family P2 that were selected for segregation analysis.

| **Gene** | **Variant ID** | **Frequency ExAC** | **Variant type** | **Amino acid change** | **Gene component** | **phyloP** | **Grantham Score** | **Polyphen2** | **SIFT score** | **MutationTaster** |
| --- | --- | --- | --- | --- | --- | --- | --- | --- | --- | --- |
| *AAED1* | chr9:99404124G>C  rs151326868 | 0.0004375 | Substitution – non-synonymous | H200D | EXON | 5.213 | 81 | 0.999 (probably damaging) | 0.04 (deleterious) | P=1  (disease causing) |
| *ATAD2* | chr8:124346225T>C | 0.0000082 | Substitution – non-synonymous | H1124R | EXON | 2.06 | 29 | 0.048  (benign) | 0.13 (tolerated) | P=0.903  (disease causing) |

**FIGURES LEGENDS**

**Figure 1.** Segregation analysis for rs151326868 (chr9:99404124G>C; *AAED1* gene) and the SNV at chr8:124346225T>C (*ATAD2* gene) in family P2. ADHD-affected individuals are depicted by black symbols, unaffected family members are shown by white symbols and individuals with unknown ADHD status are represented by a question mark in the symbol. An asterisk beneath an individual indicates that DNA was used for whole-exome sequencing analysis. Non-reference alleles are depicted in bold.

**Supplementary Figure 1.** Pedigree structure of families. ADHD patients are depicted by black symbols, unaffected family members are shown by white symbols, and individuals with unknown ADHD status are represented by with a question mark in the symbol. An asterisk (*) beneath an individual indicates that DNA was used for whole-exome sequencing analysis. A hash (#) beneath an individual indicates that genome-wide SNP data was available and that the individual was included in the linkage analyses. Pedigrees are modified to preserve confidentiality.

**Supplementary Figure 2.** Schematic overview of the study design and statistical approach. Our study included five main steps for the identification of novel ADHD genes. By combining linkage analysis and whole-exome sequencing (WES) in three multigeneration pedigrees with multiple affected individuals, we prioritized those genes emerging from linkage regions and harboring rare variants. Those 24 genes were taken forward and were jointly analyzed in gene-set analyses of IMpACT exome-chip data. Subsequently, family-wise gene-set analysis was performed, and 12 genes from family P2 were taken forward for gene-based analyses. One gene showed significant gene-based association, and a rare variant was validated.

**Supplementary Figure 3.** Genome-wide logarithm of the odds (LOD) score graph for families P1, P2, and P3 individually. The lower horizontal, black line represents a LOD score of 2 and the upper horizontal, grey line indicates the significance level at a LOD score of 3.3.

**Supplementary Figure 4.** Genome-wide logarithm of the odds (LOD) score graph for the different combinations of the three families. The lower horizontal, black line represents a LOD score of 2 and the upper horizontal, grey line indicates the significance level at a LOD score of 3.3.

**Supplementary Figure 5**. GO term enrichment analysis. To assess whether the 12 prioritized genes from family P2 converge on biological shared functions we tested for enrichment in Gene Ontology (GO) terms for biological processes using FUMA^11^. Benjamini-Hochberg correction (FDR) was used as multiple test correction method for gene-set enrichment testing. Only adjusted P-values for gene set association < 0.05 are shown.
