# Supplementary figures and images for: Identification of ADHD risk genes in extended pedigrees by combining linkage analysis and whole-exome sequencing
Source: Mol Psychiatry. 2018 Aug 16;25(9):2047–57. doi: 10.1038/s41380-018-0210-6 (PMC7473839; doi:10.1038/s41380-018-0210-6)

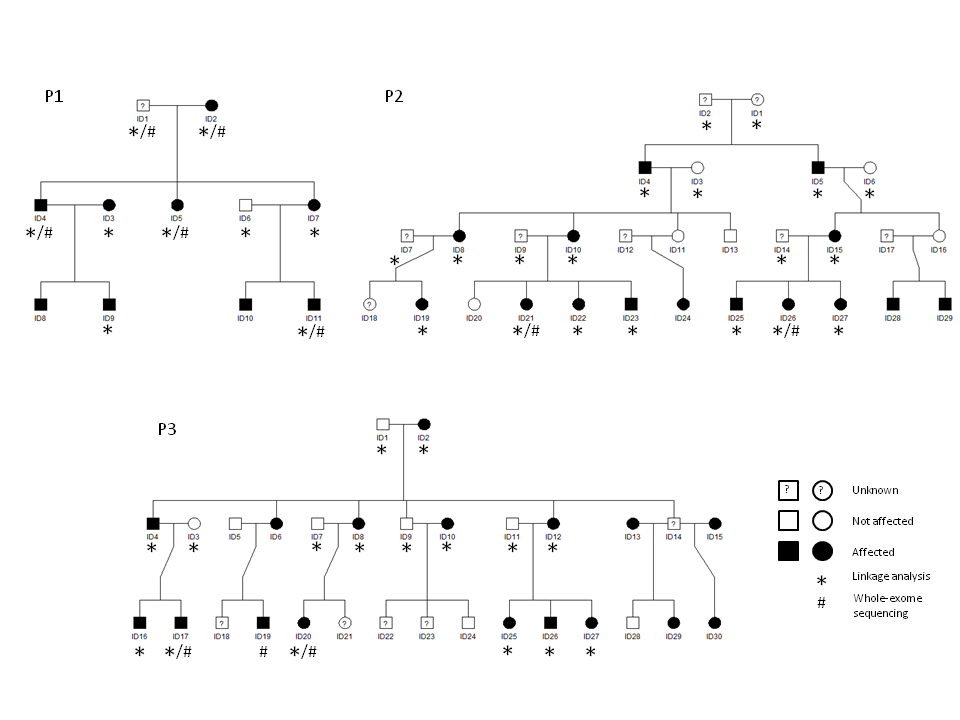

Supplement: Supplementary file 2 — Supplementary Figure 1 [file 41380_2018_210_MOESM2_ESM.png]

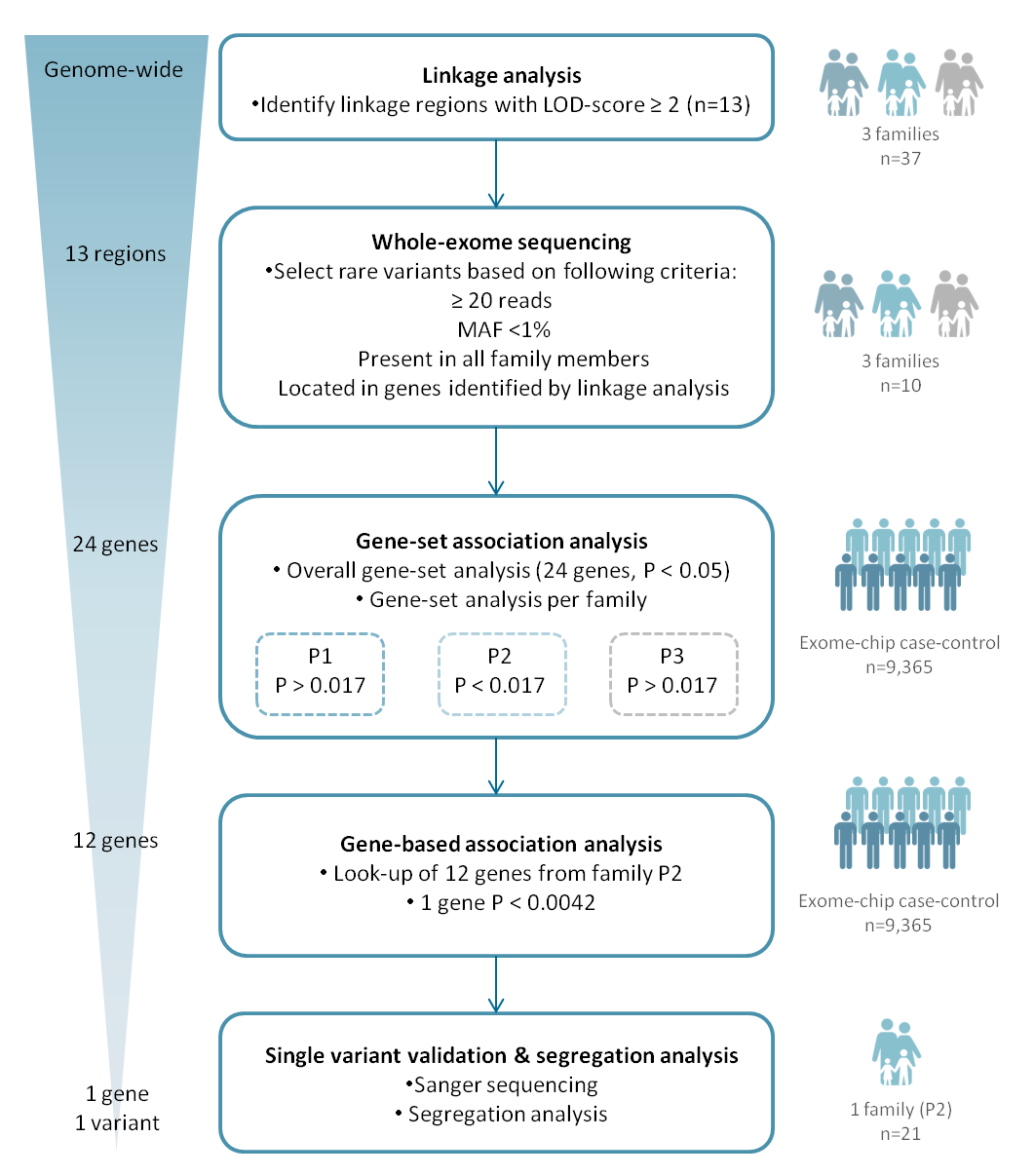

Supplement: Supplementary file 3 — Supplementary Figure 2 [file 41380_2018_210_MOESM3_ESM.png]

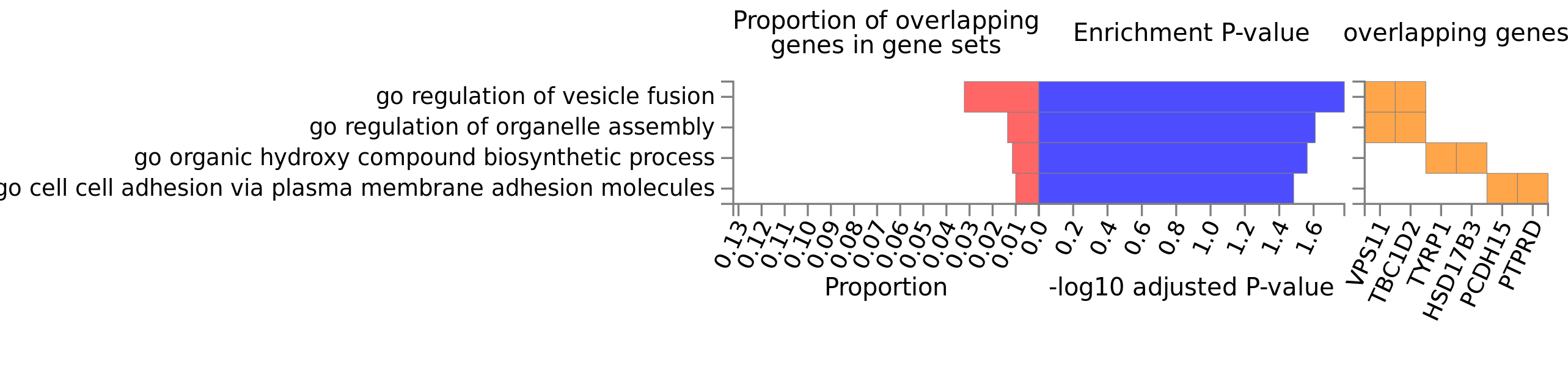

Supplement: Supplementary file 4 — Supplementary Figure 5 [file 41380_2018_210_MOESM4_ESM.png]

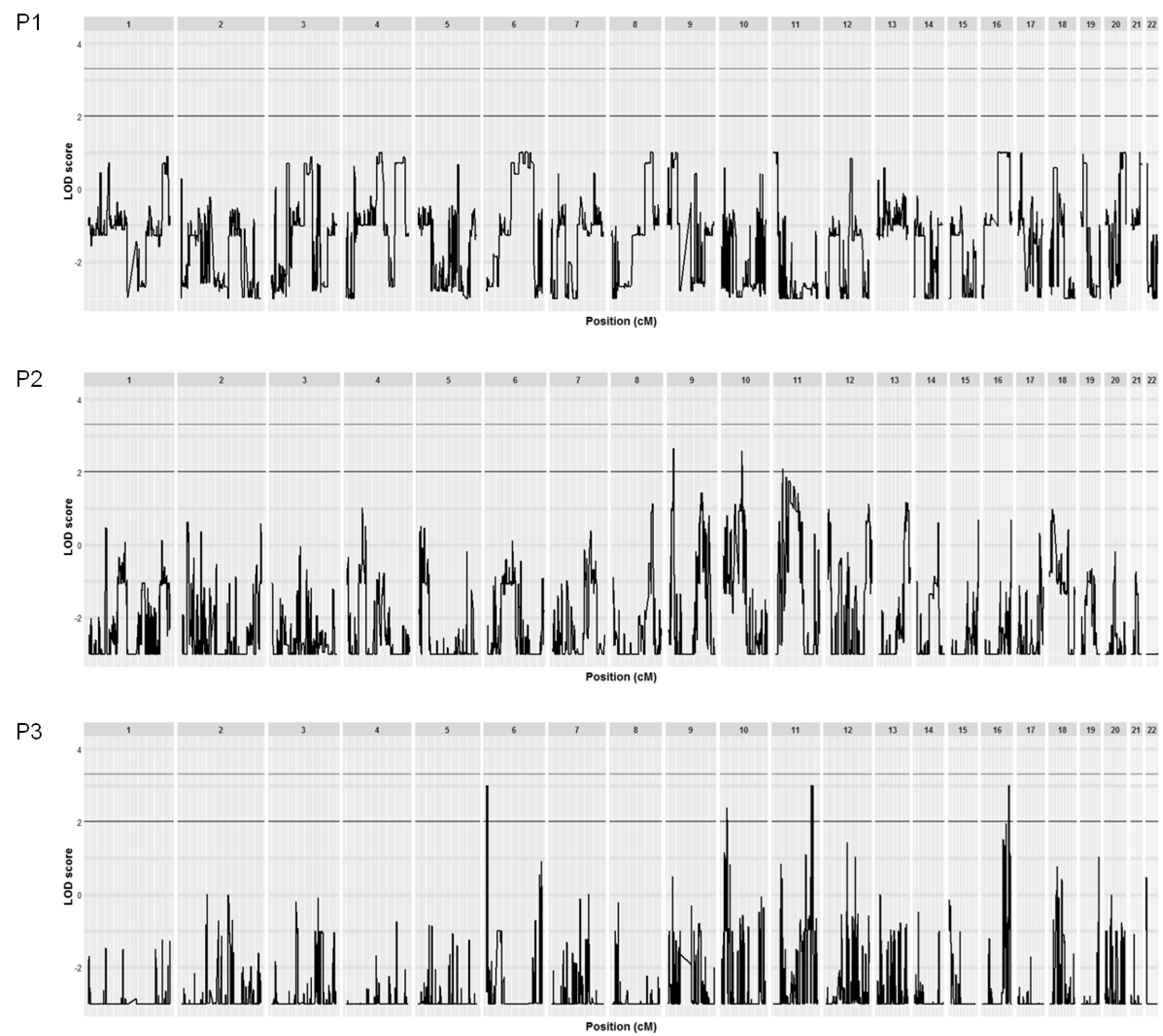

Supplement: Supplementary file 5 — Supplementary Figure 3 [file 41380_2018_210_MOESM5_ESM.tif]

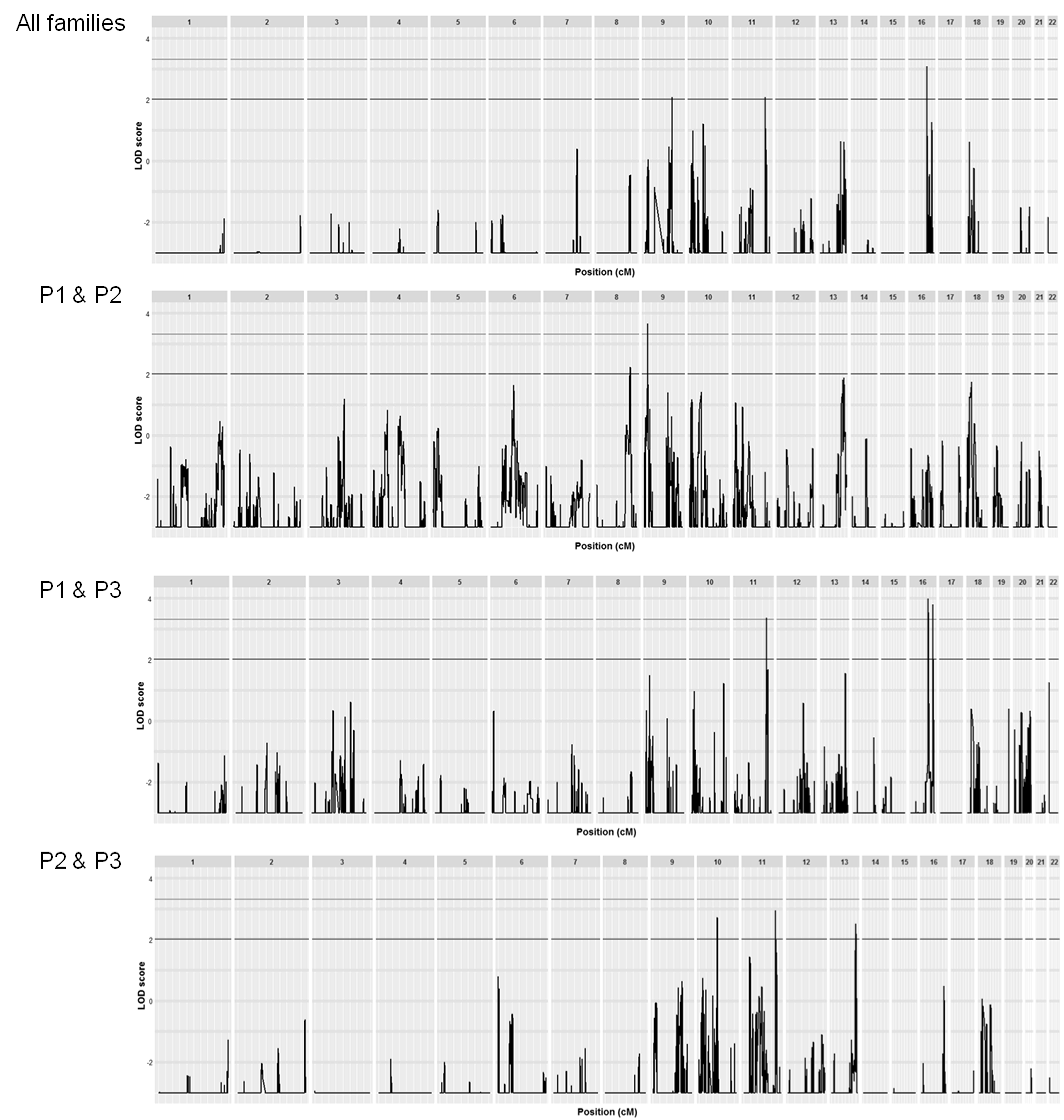

Supplement: Supplementary file 6 — Supplementary Figure 4 [file 41380_2018_210_MOESM6_ESM.tif]
